# Morphological and genetic characteristics of the novel entomopathogenic fungus *Ophiocordyceps langbianensis* (Ophiocordycipitaceae, Hypocreales) from Lang Biang Biosphere Reserve, Vietnam

**DOI:** 10.1038/s41598-020-78265-7

**Published:** 2021-01-14

**Authors:** Thuan Duc Lao, Thuy Ai Huyen Le, Nguyen Binh Truong

**Affiliations:** 1 Faculty of Biotechnology, Ho Chi Minh City Open University, Ho Chi Minh City, Vietnam; 2 Faculty of Biology, Dalat University, Dalat, Lam Dong Vietnam

**Keywords:** Fungal biology, Fungal systems biology

## Abstract

An entomopathogenic fungus newly named *Ophiocordyceps langbianensis* was collected from Lang Biang Biosphere Reserve, located in Lam Dong Province, Vietnam. It is characterized as a species of *Ophiocordyceps* (*Ophiocordycipitaceae*, *Hypocreales*) having the unique characteristics of a cylindrical fertile part and several branched apical appendices. Each ascospore develops as two swollen, constricted part-spores. A phylogenetic analysis of multiple genes, including *nrLSU*, *nrSSU*, *Rpb1*, *ITS* and *Tef,* supported its systematic position in the genus of *Ophiocordyceps*; it is related to *O. brunneipunctata*. Based on morphological and phylogenetic analyses, *O. langbianensis* was confirmed as a new species from Vietnam.

## Introduction

The genus *Ophiocordyceps*, first established by Petch in 1931, belongs to the family Ophiocordycipitaceae, order Hypocreales, comprising approximately 250 species^1,2^. Originally, *Ophiocordyceps* was classified as a subgenus of *Cordyceps* by Kobayasi (1941, 1982) and Mains (1958)^3–5^. In 2007, Sung et al. established a new called family Ophiocordycipitaceae, comprising *Ophiocordyceps,* based on morphological and phylogenetic analyses^6,7^. The distinction of the genus *Ophiocordyceps* from *Cordyceps* was done due to the darkly pigmented stromata of *Ophiocordyceps*, which are pliant, wiry or fibrous and tough in texture, compared to the brightly pigmented stromata of *Cordyceps*^7^. Species of *Ophiocordycep*s are entomopathogenic on a wide range of insects. The hosts of species of *Ophiocordyceps* are the larva*s* of *Coleoptera* and *Lepidoptera* as well as the adults of *Araneae, Diptera*, *Hemiptera*, *Hymenoptera*, *Odonata* and *Orthoptera*^3–7^. Although *Ophiocordyceps* has worldwide distribution, the tropics and subtropics are where the highest numbers of the species are recorded. Moreover, it is considered that there is an underestimation of the number of *Ophicordyceps* species.

Vietnam is located in a tropical region with terrestrial ecosystems. The forests feature a rich biodiversity of both flora and fauna due to the tropical monsoon climate with high temperature and rainfall. This is a favorable environment for the development of entomopathogenic fungi. Lang Biang Biosphere Reserve is located in Lam Dong Province and comprises a vast primitive jungle with the Lang Bian Mountain at its core, one of Vietnam’s four biodiversity centers. During our expedition to discover the diversity of entomopathogenic fungi, we collected the sample DL0017. In this study, we introduce this specimen as a new species of *Ophiocordyceps* that parasitizes the larva of *Coleoptera*. We present a morphological description and phylogenetic analysis based on the phylogenetic construction of nuclear large ribosomal subunit (nrLSU), nuclear small ribosomal subunit (nrSSU) and RNA Polymerase II Subunit B1(rpb1) of species of *Ophiocordyceps,* including this new species.

## Materials and methods

### Fungal specimen collection

The specimen, DL0017, used for this study was collected from Lang Biang Biosphere Reserve (N 12°2′19.0″, E108°26′04.7″, elevation 1680 m) in 9th August, 2016. The specimen, including the host, was extracted carefully, noted, and photographed in the field using a digital camera. The specimen was immediately wrapped in wax paper, placed in a collection bag, and taken to the laboratory.

### Cultivation techniques

According to the identification of conidia, phialides and colony coloration, the isolate cultures were grown on YMG media, composed of 4 g/l yeast extract (Sigma-Aldrich, Germany), 10 g/l malt extract (Sigma-Aldrich, Germany), 4 g/l glucose (Sigma-Aldrich, Germany), and incubated at 20 °C for a period of 20 days with PDA media (potato extract 4 g/l, dextrose 20 g/l, agar 15 g/l; Merck, Germany).

For fruit body induction, cultures were grown on millet substrate (millet/silkworm pupae powder = 20:1 (w/w)) and brown rice substrate (brown rice/silkworm pupae powder = 20:1 (w/w)) at 20 °C under 12 h light and 12 h darkness with relative humidity of over 90%.

### Morphological study: macro- and micro-morphological analysis

Morphological observations were carried out and recorded according to the guidelines of Kobayasi and Sung et al.^3,4,7^. The macroscopic characteristics of the fresh fruit body were carefully observed, including the stipe, stroma, etc. Moreover, the color was noted according to Kornerup and Wanscher^8^. Additionally, the host insect was identified based on morphological characteristics, such as mandibulate mouthparts, antennae, shape of head and thorax. For the micro-morphological analysis, one or two perithecia were removed from the stroma and placed on a microscope slide in lactophenol-cotton blue to measure the sizes and shapes of the perithecia, asci and ascospores. Finally, the nomenclatural novelty and descriptions were deposited in MycoBank.

### DNA extraction, PCR amplification, target gene sequencing

Genomic DNA was isolated by using the phenol/chloroform method (pH = 8)^11^. The fruiting body was incubated in a lysis buffer (2.0% SDS, Tris–HCl pH 8.0, 150 mM NaCl, 10 mM EDTA, 0.1 mg/ml Proteinase K) at 65 °C overnight. The supernatant was collected by centrifugation, and a volume of 700 μL of phenol/chloroform/isoamyl alcohol (25:24:1) was supplemented and centrifuged. The supernatant was collected and precipitated with absolute isopropanol. Finally, the isolated genomic DNA was stored in Tris–EDTA buffer at − 20 °C for further studies.

The primer pairs used to amplify *nrLSU*, *nrSSU*, *rpb1, ITS and Tef* regions are shown in Table 1. The final volume of PCR was done in a total of 15 μL with the thermal program: 1 cycle at 95 °C for 5 min, 40 cycles at 95 °C for 30 s, X °C for 30 s, 72 °C for 2 min, 1 cycle at 72 °C for 5 min (Note: X °C is the annealing temperatures for each target gene shown in Table 1); 5 μL aliquots of amplification product were electrophoresed on a 2.0% agarose gel and visualized in a UV transilluminator. The amplified product was sequenced at Nam Khoa (Vietnam) company.

**Table 1:** The primers’ sequence used in this study.

Target gene	Primer	Sequence (5′–3′)	Ta (^o^C)	References
*nrLSU*	LR0R (F)	GTACCCGCTGAACTTAAGC	55	^9^
	LR5 (R)	ATCCTGAGGGAAACTTC		
*nrSSU*	NS1 (F)	GTAGTCATATGCTTGTCTC	42.2	^10^
	NS4 (R)	CTTCCGTCAATTCCTTTAAG		
*Rpb1*	CRPB1 (F)	CCWGGYTTYATCAAGAARGT	55	^6^
	RPB1Cr (R)	CCNGCDATNTCRTTRTCCATRTA		
*ITS*	ITS1F (F)	CTTGGTCATTTAGAGGAAGTAA	55	^10^
	ITS4 (R)	TCCTCCGCTTATTGATATGC		
Tef	983F (F)	GCYCCYGGHCAYCGTGAYTTYAT	55	^10^
	2218R (R)	ATGACACCRACRGCRACRGTYTG		

F: Forward primer; R: Reverse primer; T_a_: Annealing temperature.

### Taxa and *nrLSU*, *nrSSU*, *rpb1*, *ITS* and *tef* sequences collection, DNA proofreading and phylogeny analysis

The data set of *nrLSU*, *nrSSU*, *rpb1, ITS* and *tef* sequences were established by sequences downloaded from Genbank (NCBI) and based on the previous data published by Sung et al.^7^. The *nrLSU*, *nrSSU*, *rpb1, ITS* and *tef* were noted with accession number, name of taxon and locality. The amplified DNA sequences were proofread to remove ambiguous signals at both ends by different software, including Seaview 4.2.12 and Chromas Lite 2.1.1. The phylogenetic tree was constructed based on neighbor-joining (NJ), maximum parsimony (MP), and maximum likelihood (ML), using Molecular Evolutionary Genetics Analysis (MEGA) version 5. Additionally, the best evolution model was predicted using jModelTest.

## Results

### Taxonomy

*Ophiocordyceps langbianensis* T. D. Lao, T. A. H. Le & N. B. Truong, sp. nov.

**Figure 1:**
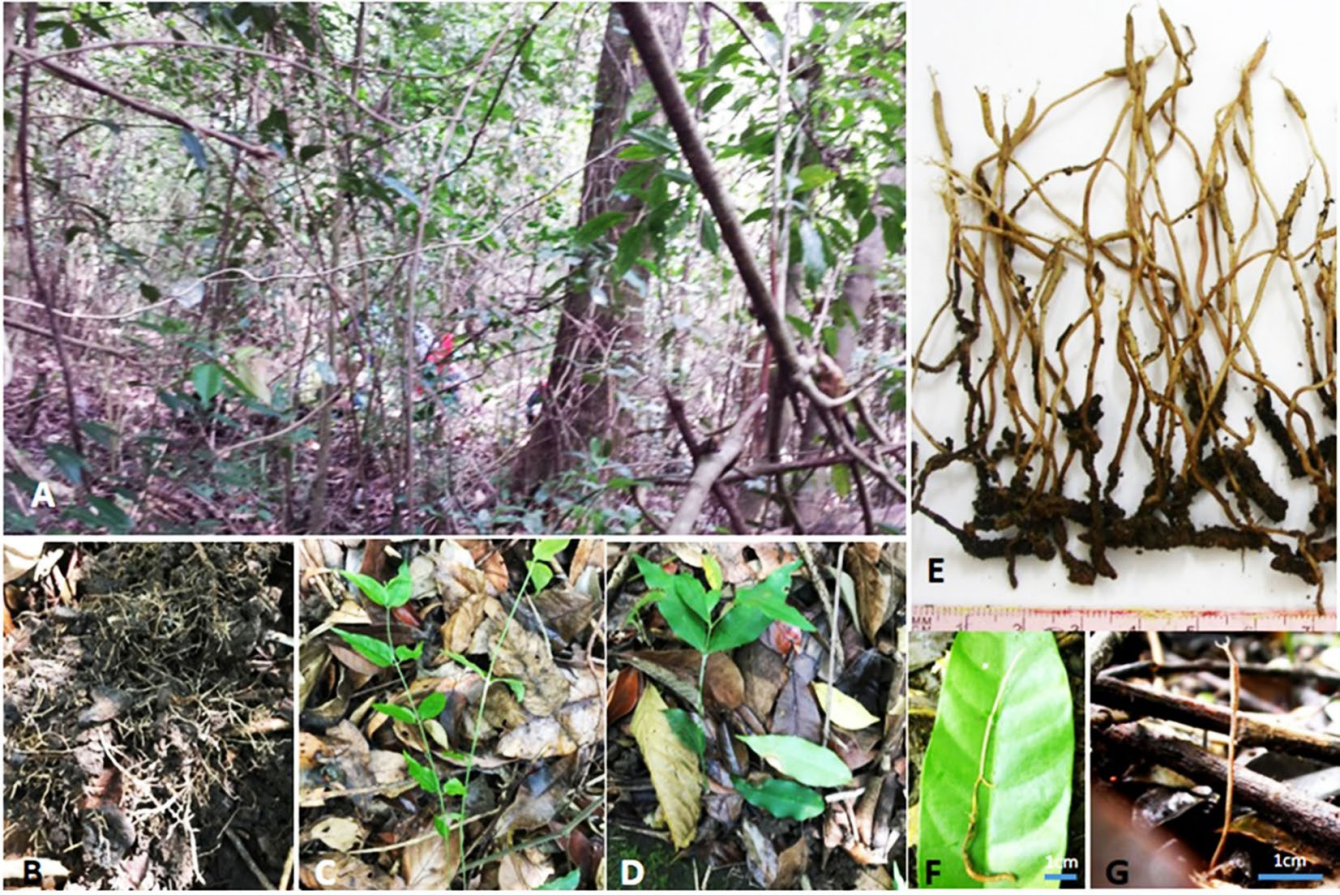
Overview of *Ophiocordyceps langbianensis*. (**A**–**D**) Ecology of collected plots; (**E**) Stroma developing from the head of hosts; (**F**) Immature stromata of fungus emerging from the larva of Coleoptera; (**G**) Stromata in moist soil surrounded by dried leaves.

**Figure 2:**
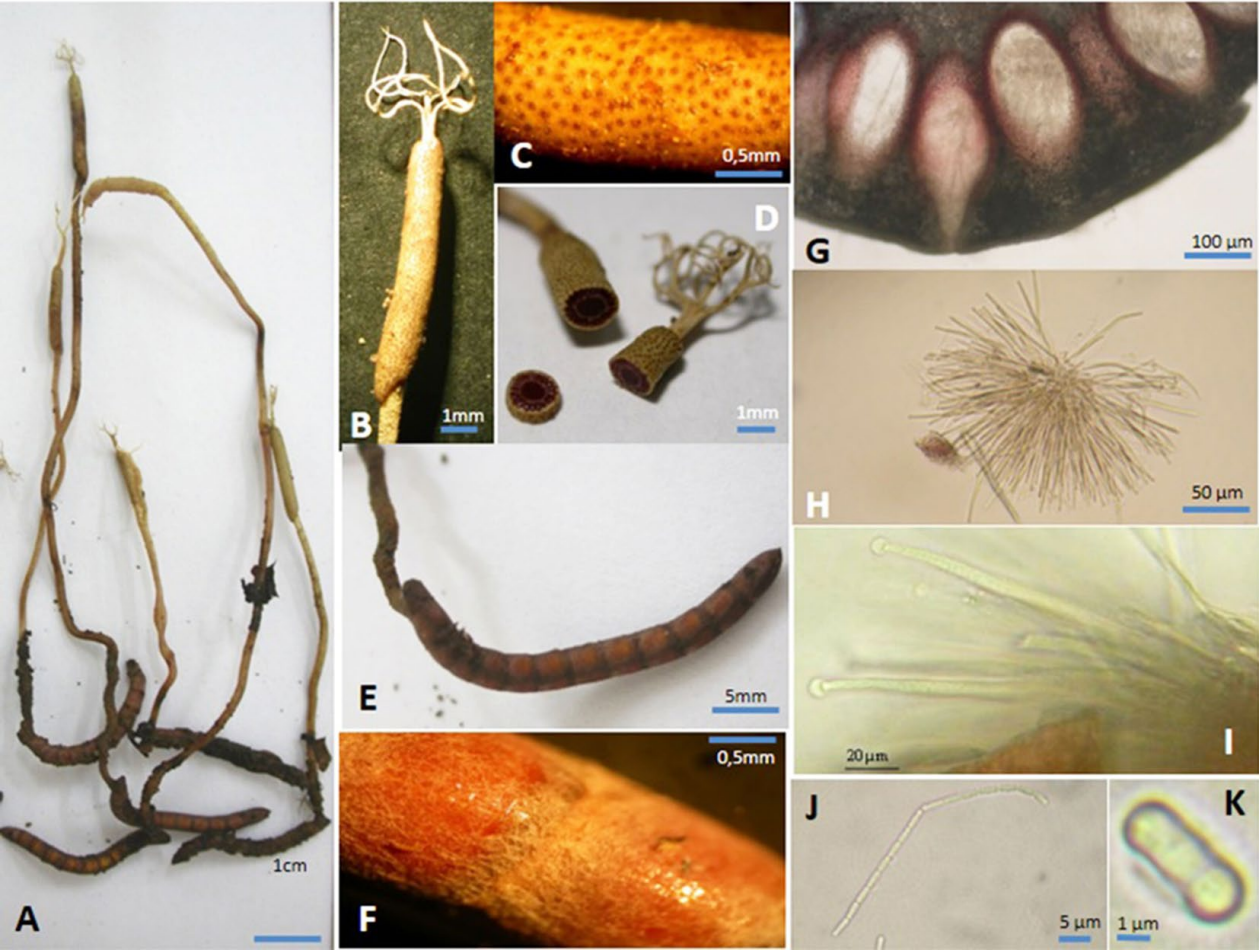
*Ophiocordyceps langbianensis*. (**A**) Stroma on host; (**B**–**D**) Fertile part and apical appendix, surface of fertile part with perithecium ostioles, cortex; (**E**) Host; (**F**) Mycelium on the host; (**G**) Perithecia; (**H**, **I**) Asci with thick cap; (**J**, **K**) Ascospores.

**Figure 3:**
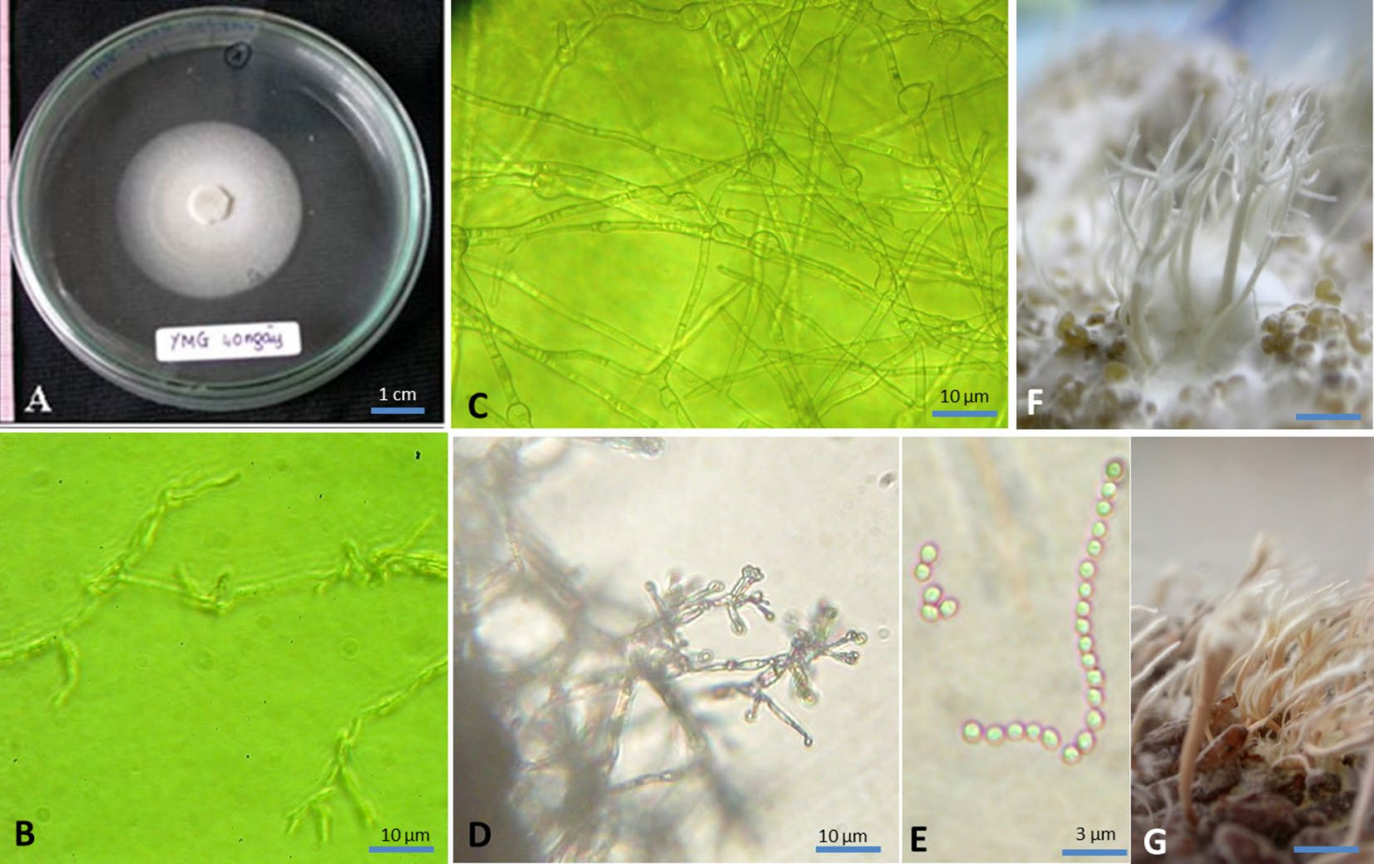
Asexual states. (**A**) Ascospores germinating after 96 h; (**B**) Septate hyphae, branched, conidia in intercalary or terminal cells; (**C**) White colony after 45 days on YMG media; (**D**) Aerial hyphae with divergent phialides; (**E**) Elliptical conidia in chains after release from the phialide, (**F**, **G**) Stromata growing on cereal substrates.

Mycobank MB836716 Figs. 1, 2, 3.

#### Typification

VIETNAM. Lam Dong Province, Lang Bian Biosphere Reserve, Lang Bian mountain: N 12°02′19.0″, E108°26′04.7″; elevation 1680 m; humidity: over 85%; temperature: day 20 °C–22 °C, night: 14 °C–16 °C; collected between 9h00–15h00 of the day on 9 August, 2016, from the larva of a beetle of *Coleoptera* in moist soil surrounded by dried leaves. Truong B.N. DL0017 (Holotype DLU; Iso VNMN, DLU).

#### Distribution

Vietnam, only known from Lang Bian Mountain.

#### Etymology

“Langbianensis” refers to Lang Bian Mountain, Lam Dong province, Vietnam.

#### Host

On the larva of a beetle of *Coleoptera*. Larva: 28–32 mm long, hard-body, shiny, smooth, dark brownish yellow; body composed of 13 segments with black edges; larva with three pairs of jointed legs attached to thorax.

#### Habitat

Individuals of associated species appeared at the type locality, including pioneer species such as *Acer laurinum* (Aceraceae)*, Baccaurea harmandii* (Euphorbiaceae)*, Castanopsis chinensis* (Fagaceae)*, Eriobotrya poilanei* (Rosaceae)*, Jasminum longisepalum* (Oleaceae)*, Phoebe petelotii* (Lauraceae) and *Tetrastigma lanceolarium* (Vitaceae)*.*

#### Sexual morph

Stroma arising from the head of the host larva, solitary, rarely branched, 40–100 mm long; host covered with thin, tough layer of mycelium. Stipe filiform, cylindrical, 30–67 mm × 0.7–1.0 mm, pale yellow. Fertile portion, cylindrical, 7.0–14.0 mm × 1.5–2.0 mm, brownish yellow with dark brown ostiolar dots of perithecia. Apical appendices, pale yellow, 2–10 primary or secondary branches, 4.0–10.0 × 0.5 mm. Perithecia immersed, ovate or pyriform, 260–400 μm × 100–190 µm. Asci, cylindrical, 200–250 μm × 5.0–6.0 μm, with thickened cap. Ascospores filiform, multiseptate, articulated in long-chain after discharging, sometimes breaking into 1-celled part spores, cylindrical, swollen, two waist-like constrictions, 5–7.5 μm × 1.3–2 µm.

#### Asexual morph

Germination of ascospores after 48 h on PDA; white colony, slow growing on YMG and PDA media, 25.00 mm and 24.58 mm after 40 days (respectively); septate hyphae, branched, chlamydospores developing in intercalary or terminal cells. Aerial hyphae with divergent phialides; elliptical conidia in chains after release from phialide. Stromata without fertile part forming on cereal substrates. Minor differences in morphological characteristics of stromata developing from different substrates. Stromata, white, branched when developing on millet substrate; brownish yellow, solitary, rarely branched, when developing on brown rice substrate.

### Amplification of *nrLSU*, *nrSSU*, *rpb1*, *ITS* and *tef* genes

Target genes, including *nrLSU*, *nrSSU*, *rpb1, ITS* and *tef*, were successfully amplified with corresponding primers (Table 1). The bands of 950-bp, 1102-bp, 803-bps, 700-bps, and 1030-bps corresponding to the amplified *nrLSU*, *nrSSU*, *rpb1*, *ITS and tef* were observed in the electrophoresis on 2.0% agarose gel. The PCR products were sequenced with the signal of the peaks in both strands of target genes; the sequence was significant, unique and good for reading.

### The systematic concatenated *nrLSU*, *nrSSU*, *rpb1*, *ITS* and *tef* gene dataset

To construct a phylogeny of major lineages, representative taxa were chosen based on previous study^7^. The data set of *nrLSU*, *nrSSU*, *rpb1, ITS* and *tef* consisted of 50, 50, 46, 39 and 42 taxa representing the morphological and ecological diversity of genera in Ophiocordycipitaceae, Clavicipitaceae, and Cordycipitaceae, including the outgroup taxon *Glomerella cingulata* (Glomerellaceae, Glomerellales) (Table 2). A combined concatenated dataset consisting of 30 representative taxa was constructed based on the list of individual target genes.

**Table 2:** Representative taxa information and GenBank accession numbers for sequences used in current study.

Taxon	Genus	*nrLSU*	*nrSSU*	*rpb1*	*ITS*	*Tef*
*Claviceps fusiformis*	Claviceps	U17402	DQ522539	DQ522366	JN049817	DQ522320
*Claviceps paspali*	Claviceps	U47826	U32401	DQ522367	JN049818	DQ522321
*Claviceps purpurea*	Claviceps	AF543789	AF543765	AY489648	KJ529004	AF543778
*Claviceps purpurea*	Claviceps	EF469075	EF469122	EF469087	KX977396	EF469058
*Metacordyceps chlamydosporia*	Metacordyceps	DQ518758	DQ522544	DQ522372	–	EF469069
*Metaccordyceps taii*	Metacordyceps	AF543787	AF543763	DQ522383	–	AF543775
*Metacordyceps liangshanensis*	Metacordyceps	EF468815	EF468962	–	–	EF468756
*Metacordyceps liangshanensis*	Metacordyceps	EF468814	EF468961	–	–	EF468755
*Conoideocrella luteorostrata*	Conoideocrella	EF468850	EF468995	EF468906	JN049859	EF468801
*Conoideocrella luteorostrata*	Conoideocrella	EF468849	EF468994	EF468905	JN049860	EF468800
*Ophiocordyceps acicularis*	Ophiocordyceps	EF468805	EF468950	EF468852	JN049820	EF468744
*Ophiocordyceps acicularis*	Ophiocordyceps	EF468804	EF468951	EF468853	GU723772	EF468745
*Ophiocordyceps apholli*	Ophiocordyceps	DQ518755	DQ522541	–	–	–
*Ophiocordyceps brunneipunctata*	Ophiocordyceps	DQ518756	DQ522542	DQ522369	GU723777	DQ522324
*Ophiocordyceps sinensis*	Ophiocordyceps	EF468827	MF403011	EF468874	JN049854	EF468767
*Ophiocordyceps stylophora*	Ophiocordyceps	EF468837	EF468982	EF468882	–	EF468777
*Ophiocordyceps stylophora*	Ophiocordyceps	DQ518766	DQ522552	DQ522382	JN049828	DQ522337
*Ophiocordyceps australis*	Ophiocordyceps	DQ518768	DQ522554	DQ522385	–	–
*Ophiocordyceps variabilis*	Ophiocordyceps	EF468839	EF468985	EF468885	–	EF468779
*Ophiocordyceps entomorrhiza*	Ophiocordyceps	EF468809	EF468954	EF468857	JN049850	EF468749
*Ophiocordyceps gracilis*	Ophiocordyceps	EF468810	EF468955	EF468858	AJ786563	EF468750
*Ophiocordyceps gracilis*	Ophiocordyceps	EF468811	EF468956	EF468859	AJ786564	EF468751
*Ophiocordyceps heteropoda*	Ophiocordyceps	AY489722	AY489690	AY489651	FJ765028	AY489617
*Ophiocordyceps heteropoda*	Ophiocordyceps	EF468812	EF468957	EF468860	JN049852	EF468752
*Ophiocordyceps nigrella*	Ophiocordyceps	EF468818	EF468963	EF468866	JN049853	EF468758
*Ophiocordyceps rhizoidea*	Ophiocordyceps	EF468825	EF468970	EF468873	JN049857	EF468764
*Ophiocordyceps rhizoidea*	Ophiocordyceps	EF468824	EF468969	EF468872	MH754720	EF468765
*Beauveria caledonica*	Beauveria	AF339520	AF339570	EF469086	HQ880817	EF469057
*Cordyceps cf. pruinosa*	Cordyceps	EF468820	EF468965	EF468868	–	DQ522351
*Cordyceps cf.pruinosa*	Cordyceps	EF468821	EF468966	EF468869	–	–
*Cordyceps cf.pruinosa*	Cordyceps	EF468823	EF468968	EF468871	–	EF468761
*Cordyceps cicadae*	Cordyceps	MH879588	MH879636	MH885438	MH93774	–
*Cordyceps cicadae*	Cordyceps	MK761212	MK761207	MF416653	MH937742	–
*Cordyceps kyusyuensis*	Cordyceps	EF468813	EF468960	EF468863	–	–
*Cordyceps militaris*	Cordyceps	AY184966	AY184977	DQ522377	–	DQ522332
*Cordyceps pruinosa*	Cordyceps	AY184968	AY184979	DQ522397	–	EF468763
*Cordyceps scarabaeicola*	Cordyceps	AF339524	AF339574	DQ522380	JN049827	DQ522335
*Cordyceps staphylinidicola*	Beauveria	EF468836	EF468981	EF468881	–	EF468776
*Lecanicillium antillanum*	Lecanicillium	AF339536	AF339585	DQ522396	MH861888	DQ522350
*Lecanicillium fusisporum*	Lecanicillium	AF339549	AF339598	EF468889	–	EF468776
*Lecanicillium psalliotae*	Lecanicillium	AF339559	AF339608	EF468890	–	–
*Lecanicillium tenuipes*	Lecanicillium	AF339526	AF339576	DQ522387	JN036556	DQ522341
*Cordyceps ninchukispora*	Cordyceps	EF468846	EF468991	EF468900	–	EF468795
*Cordyceps ninchukispora*	Cordyceps	EF468847	EF468992	EF468901	–	EF468794
*Simplicillium lamellicola*	Simplicillium	AF339552	AF339601	DQ522404	MH854806	DQ522356
*Simplicillium lanosoniveum*	Simplicillium	AF339554	AF339603	DQ522405	–	
*Simplicillium lanosoniveum*	Simplicillium	AF339553	AF339602	DQ522406	–	DQ522357
*Simplicillium obclavatum*	Simplicillium	AF339517	AF339567	–	MH860859	DQ522358
*Glomerella cingulate*^a^	Colletotrichum	AF543786	AF543762	AY489659	FJ904831	AF543773
*Glomerella cingulate*^a^	Colletotrichum	U48428	U48427	DQ858454	EU520087	AF543772

–: no accession number recorded.

^a^Outgroup.

### Molecular phylogeny analysis

The sequences of *nrLSU*, *nrSSU*, *rpb1*, *ITS* and *tef* of DL0017 were similar to the representative sequence of *Cordyceps brunneipunctata* (similarity > 90%), with accession numbers of DQ518756, DQ522542, DQ522369, GU723777 and DQ522324. Sequences were aligned and edited using the MEGA 5.2. Gaps were excluded from the phylogenetic analysis. The dataset of representative taxa and DL0017 target gene sequence consisted of 451 bp for *nrLSU*, 674 bp for *nrSSU*, 392 bp for *Rpb1*, 158 bp for *ITS* and 790 bp for *tef*. The evolution model that was most fixed with *nrLSU*, *nrSSU*, *Rpb1*, *ITS and tef* were TN93 + G, K2 + G + I, T92 + G + I, K2 + G, and TN93 + G + I respectively. The phylogenetic trees were generated with Neighbor Joining (NJ), Maximum Parsimony (MP), and Maximum Likelihood (ML) methods with replication of 1000. Based on the NJ, MP, and ML phylogenetic trees, individual *nrLSU*, *nrSSU*, *Rpb1*, *ITS*, and *tef* of DL0017 clustered together with *Ophiocordyceps brunneipunctata* within separate branches with credible bootstrap (≥ 50%), suggesting that these species are related (Table 3).

**Table 3:** DL0017 clustered together with *Ophiocordyceps brunneipunctata* with bootstrap support.

Gene	Bootstrap value (NJ/MP/ML)
*nrLSU*	
*nrSSU*	
*Rpb1*	
*ITS*	
*Tef*	

**Figure 4:**
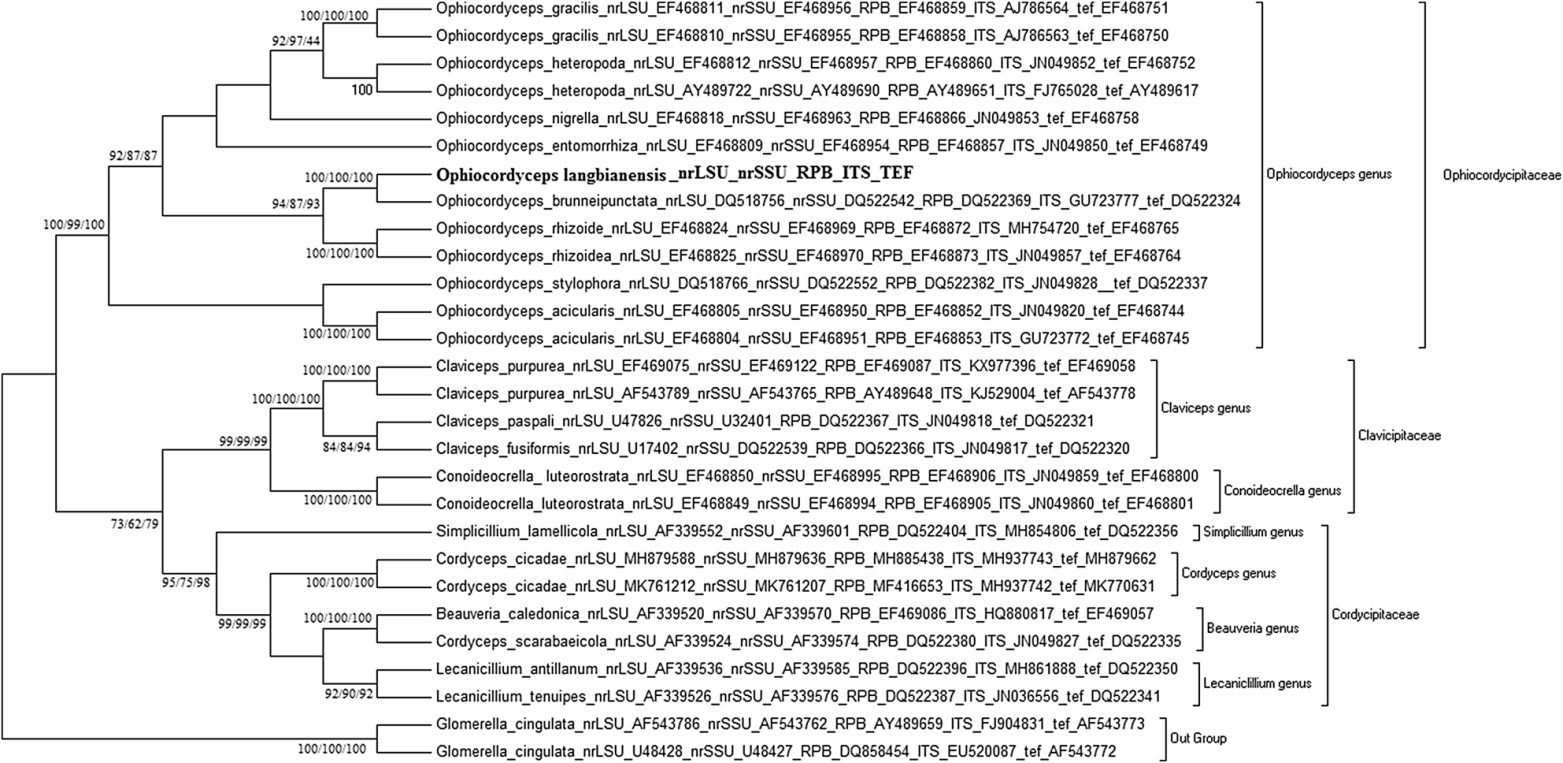
Phylogenetic relationship between *O. langbianensis* and its allies based on five regions, *nrLSU*-*nrSSU*-*Rpb*-*ITS-tef* data. Bootstrap values (1,000 replicates) are indicated above the nodes.

Information from molecular phylogenetic analysis based on separate genes is not enough to reconstruct trees for higher classification compared to multigene analysis. Therefore, a combined data set, including 2,319 bp of five target genes, *nrLSU*-*nrSSU*-*Rpb1*-*ITS-tef,* was analyzed. The evolution model that was most fixed with the combined dataset was TN93 + G + I, as determined by MEGA 5.2. The phylogenetic trees, based on analysis of the combined data, could be broadly separated into three groups, which corresponded to the families of Clavicipitaceae, Ophiocordycipitaceae and Cordycipitaceae. In the phylogenetic tree, DL0017 clustered with *Ophiocordyceps brunneipunctata* with bootstraps of 100/100/100 (NJ/MP/ML phylogenetic tree) and formed a separate, monophyletic branch. Within this monophyletic branch, DL0017 and *O. brunneipunctata* clustered together closely, suggesting that these species were truly associated (Fig. 4). The molecular phylogenetic analysis confirmed that there were differences between DL0017 and other related species.

**Figure 5:**
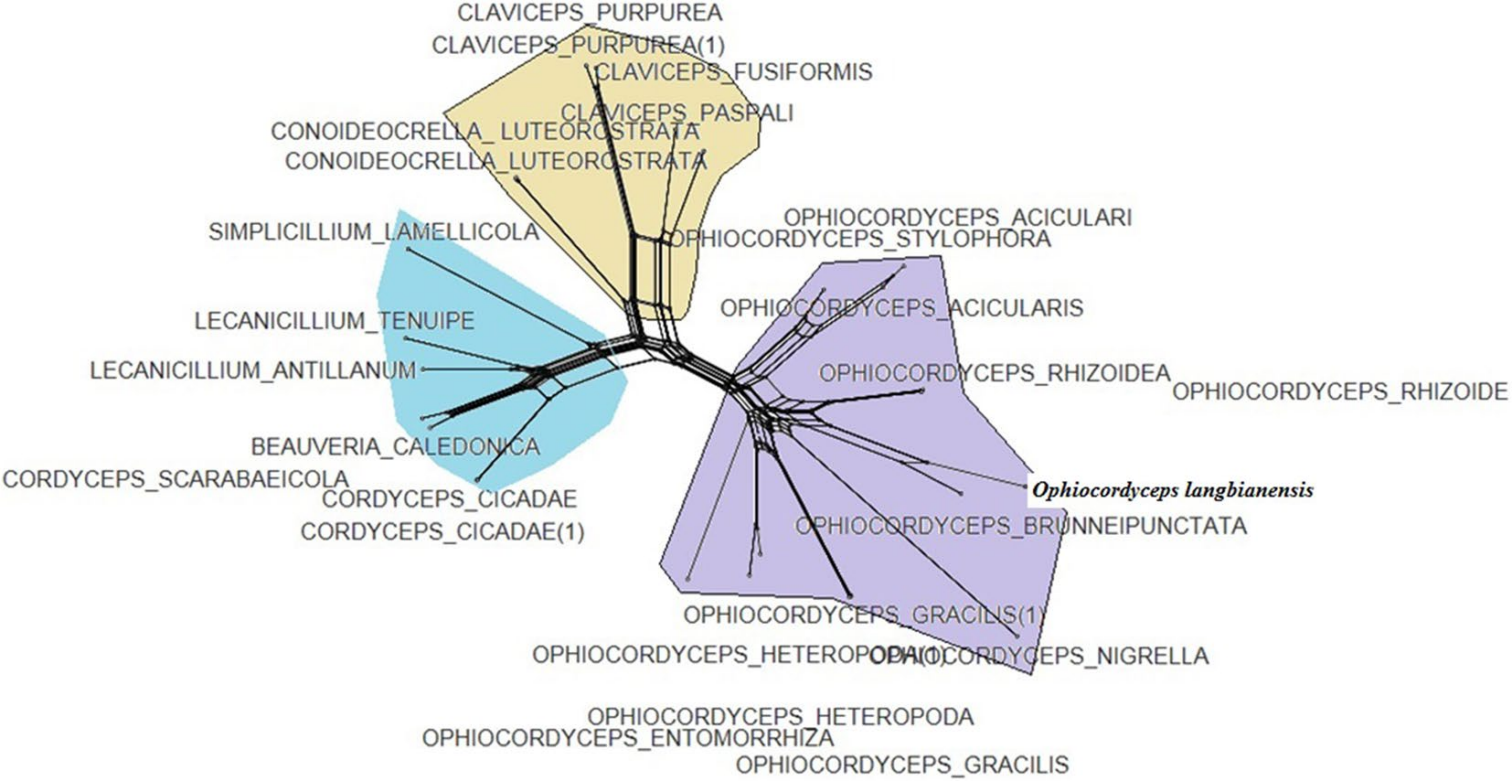
Reconstruction of Neighbor-Net network of DL0017 and its allies.

To confirm the authenticity of DL0017 as the most closely associated with *Ophiocordyceps brunneipunctata*, the reconstruction of Neighbor-Net network of DL0017 and its allies was performed. The Neighbor-Network analysis supported the results from the phylogenetic analysis (Fig. 5). The network presented three complex groups, corresponding to three families: Clavicipitaceae, Ophiocordycipitaceae and Cordycipitaceae. The DL0017 closely clustered with Ophiocordyceps complex. Additionally, speciation was observed between the cluster of DL0017 and *O. brunneipunctata*.

### Comparison of *Ophiocordyceps langbianensis* with close species

In the phylogenetic analysis, the *Ophiocordyceps langbianensis* clustered with *Ophiocordyceps brunneipunctata* with high bootstrap support, suggesting a close relationship. To confirm the authenticity of DL0017 as a new species, we compared DL0017 and its close species, *O. brunneipunctata*. It differed from *O. brunneipunctata* by the morphological characteristics described in Table 4. Therefore, DL0017 was confirmed as a new species, namely *O. langbianensis.*

**Table 4:** Comparison between *Ophiocordyceps langbianensis* và *Ophiocordyceps brunneipunctata.*

	*Ophiocordyceps langbianensis*	*Ophiocordyceps brunneipunctata*^a^
Stromata	Arising from the head of host larva Solitary, rarely branch, 40–100 mm long	Arising from one end of the insect larva Solitary, rarely up to 3, simple, 25–90 mm long
Stipe	Fibrous, cylindrical 30–67 mm × 0.7–1.0 mm, light yellow	Simple, cylindric, 5–15 mm × 1–1.8 mm, base reddish-brown
Fertile portion	cylindrical 7.0–14.0 mm × 1.5–2.0 mm, brownish yellow with dark brown dots, that present in the ostiole of the perithecia	Subterminal, cinnamon in color, with brown ostioles apparent, 5–15 × 1–1.8 mm
Perithecia	Embedded, ovate or pyriform, 260–400 μm × 100–190 µm	Immersed, ovate to pyriform, brown, 270–335 μm × 110–160 μm
Asci	Cylindric, 200–250 μm × 5.0–6.0 μm, with thick cap	Hyaline, cylindrical, 280–295 μm × 6–7 μm, with prominent apical cap
Ascospores	Filiform, multiseptate, disarticulating into unicellular partspores Partspores: cylindric, swollen, two waist-lilce constriction, 5.0–7.5 μm × 1.25–2.0 µm	Hyaline, filiform, flexuous, breaking into partspores Partspores truncate, 4–6 μm × 1–1.5 μm

^a^Reference from *Ophiocordyceps brunneipunctata* (Hywel-Jones) G.H. Sung, J.M. Sung, Hywel-Jones & Spatafora.

## Discussion

Lang Biang Biosphere Reserve, located in Lam Dong Province, is classified as Vietnam’s biodiversity center and considered a hotspot of fungal biodiversity, including entomopathogenic fungi. During our expedition to validate the diversity of entomopathogenic fungi in Lang Biang Biosphere Reserve, the sample DL0017 was collected.

Morphological analysis indicated that DL0017, named *Ophiocordyceps langbianensis,* is a new taxon. Species belonging to the family *Ophicordycipitaceae* have stromata that are darkly pigmented or rarely brightly colored), tough, fibrous, pliant, and rarely fleshy. Additionally, asci are usually cylindrical with thickened ascus apex. Ascospores are usually cylindrical, multiseptate, and disarticulate into part-spores or non-disarticulating^7^. Our specimen shares these common characteristics.

Based on the phylogenetic analysis, the specimen DL0017 clustered with *Ophiocordyceps brunneipunctata* in Ophiocordycipitaceae^12^*.* However, the morphologies of these two species are different in many characteristics, including color, size of stroma, stipe, and dots in the fertile portion. The apical appendix of *O. brunneipunctata* lacks branching, while *O. langbianensis* has 2–10 branches. Additionally, the ascospores of *O. brunneipunctata* break into part-spores, while the ascospores of *O. langbianensis* stick together to form a multiseptate chain, separating into unicellular part-spores under a strong interaction force. Multiple gene sequences of the related *Ophiocordyceps* species were used in the phylogenetic analysis. A comparison was done among the species listed in Table 4 with respect to cylindrical fertile portion, embedded perithecia, and an apical appendix. Among them, only species of *Cordyceps furcicaodata* have a branch-forming apical appendix. In the comparison between *Cordyceps furcicaodata* and *O. langbianensis*, *Cordyceps furcicaodata* was found to be smaller than *O. langbianensis*. The stroma of *Cordyceps furcicaodata* arose from the middle of the host larva, while that of *O. langbianensis* arose from one end of the insect larva^13^. As mentioned above, ascospores of *O. langbianensis* stick together to form a multiseptate chain, which could only be ruptured into unicellular part-spores by a strong force, while ascospores of *Cordyceps furcicaodata* often break into unicellular part-spores.

The asexual morph of *O. langbianensis* consists of long and divergent phialides, elliptical conidia usually in chains considered paecilomyces-like or purpureocillium-like^14,15^. Conversely, *O. bruneipunctata* produced a mononematous hirsutella-like asexual morph from colonies after 3–4 weeks.

## Conclusion

We successfully applied morphological characterization in combination with phylogenetic analysis of multiple genes, including *nrLSU*, *nrSSU*, *rpb1, ITS,* and *Tef,* to delimit sample DL0017, collected from Lang Biang Biosphere Reserve located in Lam Dong Province, Vietnam, as a new species named *Ophiocordyceps langbianensis*, belonging to the genus of *Ophiocordyceps* (Ophiocordycipitaceae, Hypocreales).
